# Mechanical and Thermal Properties of Mixed PE Fractions from Post-Consumer Plastic Packaging Waste

**DOI:** 10.1021/acsomega.2c05621

**Published:** 2022-12-01

**Authors:** Ezgi C. Boz Noyan, Abhijit Venkatesh, Antal Boldizar

**Affiliations:** Department of Industrial and Materials Science, Chalmers University of Technology, Gothenburg 41296, Sweden

## Abstract

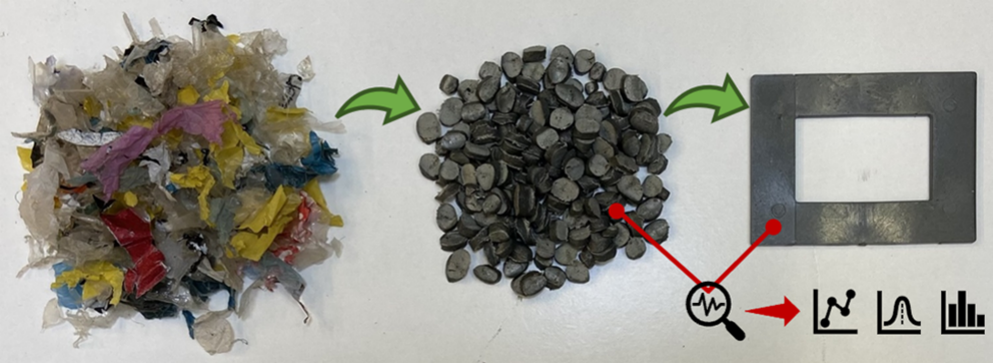

The functional properties of recycled post-consumer flexible polyethylene packaging waste have been studied using materials collected and sorted at a large-scale facility in Sweden. The studied fraction was used both as received and after simple laboratory washing in water with added sodium hydroxide at 40 °C. The materials were melt-compounded with a twin-screw extruder using two different temperature profiles and two screw configurations and injection-molded into slabs, whose thermal and mechanical properties were assessed. The results showed that the mechanical properties of injection-molded samples were not changed significantly either by the washing or by the temperature or screw configuration used in the compounding. Washing reduced the viscosity and molecular mass to a minor extent. As expected, the ash content of the compounded pellets was reduced by washing. The thermo-oxidative stability decreased with increasing compounding temperature and with washing.

## Introduction

Over the last few decades, the disposal of plastic waste has become a greater concern in the society. Recycling has generally been considered one of the favored solutions to improve plastic waste management, particularly with regard to packaging plastics. Although packaging plastics have been recycled for a long time, the recycling rates reported are still quite low. The current recycling targets in the EU regarding plastic packaging waste, set by Directive (EU) 2018/852, are 50% by 2025 and 55% by 2030.^1^ It was estimated that the rate of recycling of post-consumer plastic packaging waste (pcppw) in EU27 was ca. 14% in 2017,^2^ indicating an urgent need for an increase in plastic recycling and a better understanding of the influence of the recycling process on the properties of products made of recycled plastic waste.

Some previous studies have indicated that the addition of the recycled material influences the properties of virgin polymers and that the density of polyethylene (PE) is important.^3^ Sánchez-Soto et al.^4^ investigated the use of the heterogeneous mixed plastic waste rejected at the sorting step and reported that the properties of the mixed fraction increased with blending of either the low-density PE (LDPE) or the high-density PE (HDPE). Several studies have explored the effect of the source (e.g., mixed municipal waste and household waste) on the properties of the recyclates,^5,6^ and the effects of impurities and washing were investigated in several studies.^7−9^ Möllnitz et al.^7^ reported that washing improved several properties of the recyclate, while Streit et al.^9^ showed the importance of the choice of washing agent formulation. However, to the best of our knowledge, the influence of relevant processing parameters on the reshaped material has not been reported on sufficiently.

In a general perspective, it can be argued that little is reported on the mechanical properties of unwashed and non-treated post-consumer flexible PE packaging waste and also that few studies concern the influence of washing on the resulting product properties. As the pcppw must be expected to be heterogeneous, both in composition and in the aging status, the aspects of molecular degradation may be of high interest.

Against this background, the aim of the present study was to explore the effect of processing temperature and screw design on the physical, thermal, and mechanical properties of the recycled post-consumer flexible PE (PE-2D) waste material, collected and sorted on a large scale in Sweden. The PE fraction was selected because it is widely used for packaging films^10,11^ and is therefore one of the largest sorted fractions.^12^ The influence of washing was also assessed as several studies^7,13−18^ have shown that the purity of the sorted fraction is important and the importance of washing has been pointed out for, for example, high-quality recyclates,^13^ efficient removal of contaminates,^9,19−21^ and odor removal.^22^ NaOH solution was chosen for washing because it is known to be the commonly used washing agent in mechanical recycling.^20^ On the other hand, although it has not been extensively studied in the literature, one study reported that washing with NaOH accelerated the degradation of recycled HDPE possibly due to the created functional groups causing chain scission being more dominant.^23^

## Materials and Methods

Sampling was performed at a pcppw sorting facility, Swedish Plastic Recycling (SPR), in Motala, Sweden, in order to investigate the content of different polymer fractions, the sorted fraction of interest being the flexible PE packaging (PE-2D) fraction. Currently, SPR is the only fully automated sorting plant that receives household plastic packaging waste collected from recycling stations and curbside collection sites in Sweden. Sampling was done on successive days during 3 weeks of a 5 week period, with two intermediate weeks, specifically during weeks 47, 49, and 51 in 2020. On the end sampling day, one bale containing about 600 kg of sorted fraction was randomly selected, and a 200 L sample was taken. This gave eight bags of sorted plastic pieces for each fraction, and 100 pieces of plastic from each bag were identified using a hand-held near-infrared spectroscopy analyzer, type microPHAZIR—Thermo Scientific. The second sorted PE-2D bale taken in April 2021 was used for the property studies.

Some of the shredded flakes were washed to investigate the effect of washing on the melt processability and on the mechanical and thermal properties of the material. The shredded flakes were first soaked in water with mild agitation, at a solid-to-liquid ratio of 1/60, and this was followed by a washing, rinsing, and spinning cycle. Washing was carried out in a Vortex M6, SDL Atlas (USA) machine at 40 °C using NaOH as the washing agent. The solid-to-liquid ratio (S/L) was kept at 1/40, and the amount of NaOH used was 20 wt % with respect to the amount of the sample. Washing and rinsing were carried out for a duration of 15 min each with rinsing at 25 °C. The total washing time, including the draining, re-filling, and spinning cycle, was around 40 min, and 1 kg of the material was washed per batch. Washing was followed by drying for at least 24 h at 60 °C.

**Figure 1:**
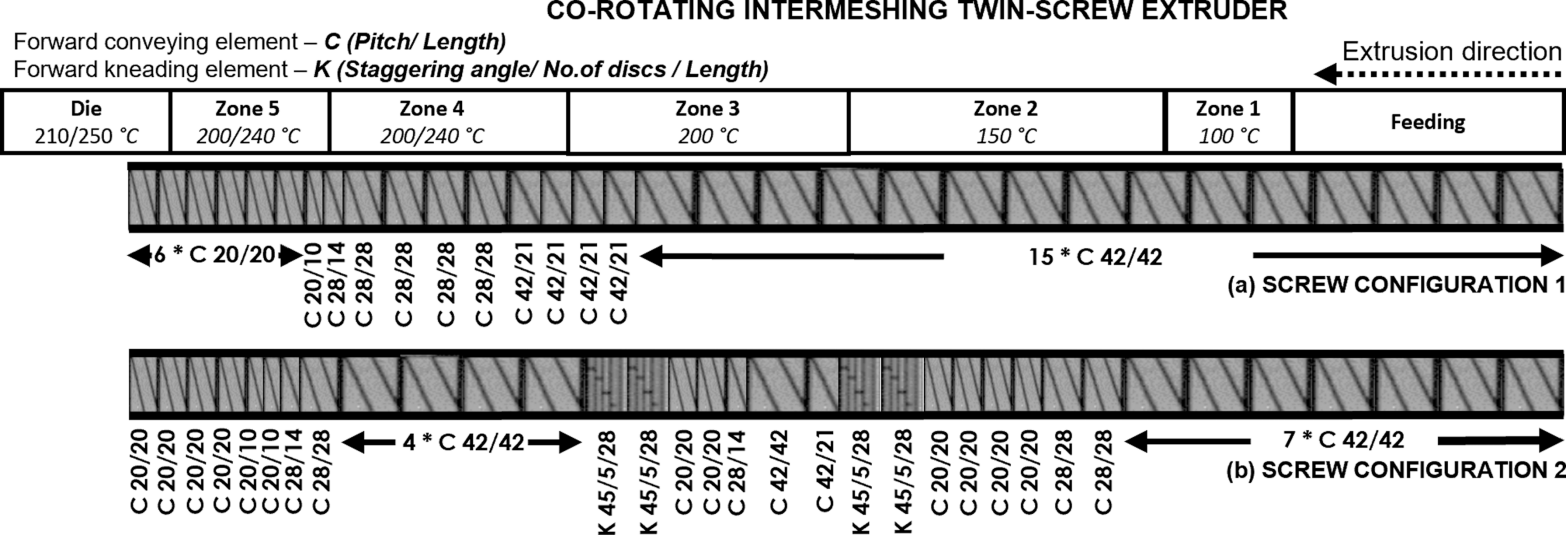
Co-rotating intermeshing TSE screw configurations used for compounding: (a) screw configuration 1: no mixing elements and (b) screw configuration 2: with four mixing elements.

The compounding was performed using a Werner & Pfleiderer ZSK 30 M9/2 co-rotating intermeshing twin-screw extruder (TSE) having a screw length of 969 mm and a diameter of 30 mm. To evaluate the influence the temperature profile used in the compounding, profiles of 100–150–200–200–200–210 °C and 100–150–200–240–240–250 °C were chosen. Two different screw configurations were used to investigate the effect of mixing, as shown in Figure 1. The first screw configuration (SC1) had only transport elements, whereas the second configuration (SC2) had four mixing elements per screw shaft. The unwashed flakes were screened before compounding using a magnetic grid to remove metal particles. The washed samples did not require magnetic screening due to the separation achieved in the pre-soaking. All the materials were fed manually into the extruder at a rate of 1.4 ± 0.4 kg/h. The screw rotation rate was 80 rpm. The compounding yielded pellets used for injection molding, see Table 1.

**Table 1:** Sample Codes with the Corresponding Processing Details

	compounding parameters		
washing status	screw design	*T* profile (°C)	sample code
unwashed	SC1	100–150–200–200–200–210	SC1_200
unwashed	SC1	100–150–200–240–240–250	SC1_240
unwashed	SC2	100–150–200–200–200–210	SC2_200
unwashed	SC2	100–150–200–240–240–250	SC2_240
washed (NaOH-40 °C)	SC2	100–150–200–200–200–210	NaOH40_SC2_200
washed (NaOH-40 °C)	SC2	100–150–200–240–240–250	NaOH40_SC2_240

**Figure 2:**
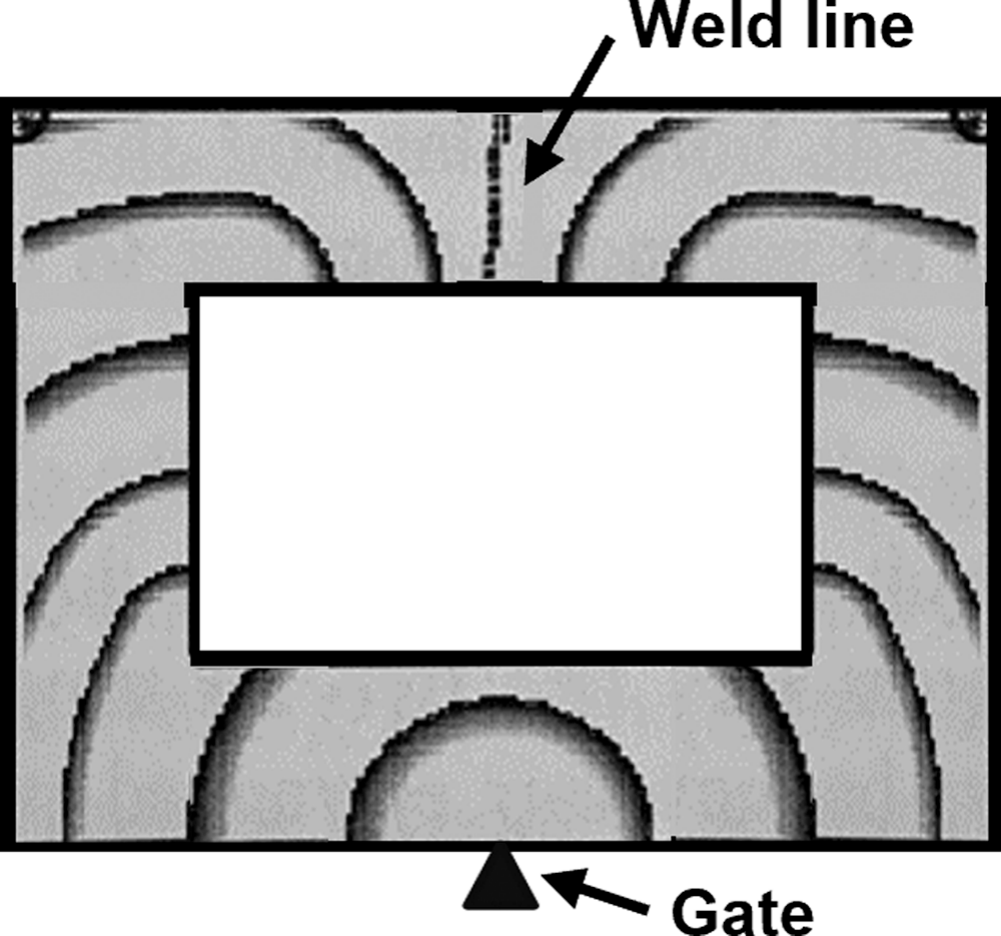
Filling pattern in the frame mold; molded samples having an overall width of 64 mm, a length of 48 mm, and a thickness of 2 mm.

An Arburg Allrounder 221M-250-5 machine was used for the injection molding, and the samples molded had the shape of a frame, as shown in Figure 2, in order to make it possible to assess the mechanical properties of different material structures in the gate region (G) with a mixed state of molecular orientation, in the simple flow region (SF) having mainly unidirectional flow, and in the weld line region (WL) where two flow fronts meet. These different structures commonly occur in conventional products. The processing parameters used in the injection molding of the frame samples were a temperature profile of 120, 170, 200, 220, 220 °C and injection and holding pressures of 500 and 700 bar, respectively. The injection volume was adjusted for each material type to achieve at least an 80% meeting of the weld line width before the holding pressure was applied.

The compounding and molding steps were applied to both unwashed and washed sorted PE-2D fractions. Table 1 shows the sample codes used in the rest of the paper.

A Mettler Toledo DSC 2 was used to determine the oxidation induction temperature (*T*_ox_) and the thermal transitions of the recycled materials according to ISO 11357-6 and ISO 11357-1, respectively. The thermal characterizations were performed on both compounded pellets and injection-molded samples. Circular sections, with a thickness of 0.65 ± 0.1 mm, were prepared for the *T*_ox_ measurements, and the thermal transitions were assessed on samples with a weight of at least 5 mg. Air was used for purging when determining the *T*_ox_ and nitrogen when determining the thermal transitions, both at a heating rate of 10 °C/min. Duplicate measurements were made on each type of material, and the mean values were calculated. For the measurement of change in enthalpy Δ*H*, the baseline was taken from 60 to 132 °C.

The ash content of the samples was measured with a TGA/DSC 3+ Star system from Mettler Toledo. The pellets were ground into a powder, and 3 ± 1 mg of sample was heated from 25 to 650 °C at a rate of 10 °C/min in air at a flow rate of 50 mL/min. Duplicate measurements were made on each type of material, and the mean values were calculated.

The melt mass-flow rate (MFR) values of the pellets were determined using a Ceast modular melt flow (Instron) instrument with a standard weight of 2.16 kg at 190 °C in accordance with ISO 1133-1:2011.

The molecular weights of selected samples were assessed by high-temperature gel permeation chromatography at ITS Testing Services (UK) Limited (Redcar, UK). Samples were dissolved at a concentration of 4 mg/mL in 1,2,4-trichlorobenzene with 200 ppm BHT as an antioxidant. The analyses were performed using a Polymer Laboratories GPC220 instrument with PlOlexis and PlOlexis guard columns with lengths of 3 × 30 cm at 160 °C, with an injection volume of 200 μL and a flow rate of 0.8 mL/min. Data were captured and analyzed using Polymer Laboratories Cirrus software. The results shown are the weight-average molecular mass (*M*_w_) and polydispersity index (PDI) based on two independent measurements.

The rheological behavior of selected samples was studied using pellets obtained after compounding in a high-pressure capillary rheometer Rheograph 20 (Göttfert) at 220 °C using a constant piston speed at each shear rate between 10^3^ and 10^1^ s^–1^. Three dies were used having a diameter of 2 mm and aspect ratios (*L*/*D*) of 5, 10, and 15 for the Bagley correction with respect to the ISO standard 11443:2021. A Weissenberg–Rabinowitsch correction was applied according to ISO 11443:2021. The graphical results show the corrected viscosity (η) versus the shear rate (γ̇), assessed with the die with a *L*/*D* ratio of 10.

The tensile properties were measured with a Zwick/Z2.5 instrument equipped with a 2 kN load cell. Test bars were cut from the three different regions of the molded frame using an Elastocon EP 04 ISO 37-2 cutting die, corresponding to specimen-type 5A in ISO 527-2, and kept in a conditioned environment of 50 ± 10% relative humidity and 23 ± 2 °C for at least 24 h prior to the tensile tests. The tensile properties measured were the Young’s modulus, tensile strength, and strain at break at a strain rate of 1 s^–1^. The reported average values and standard deviations are based on five independent measurements.

The thermal decomposition products which evolved were determined by thermogravimetry-coupled FTIR using a TGA 2 (Mettler Toledo) with a Nicolet iS50 FTIR (Thermo Fisher Scientific) attachment. The unwashed sample (3 mg) compounded with SC1 at 200 °C was first heated from 25 to 600 °C at a rate of 10 °C/min in nitrogen and then from 600 to 800 °C at a rate of 20 °C/min in air, and the resulting spectra were analyzed with OMNIC software.

## Results and Discussion

**Figure 3:**
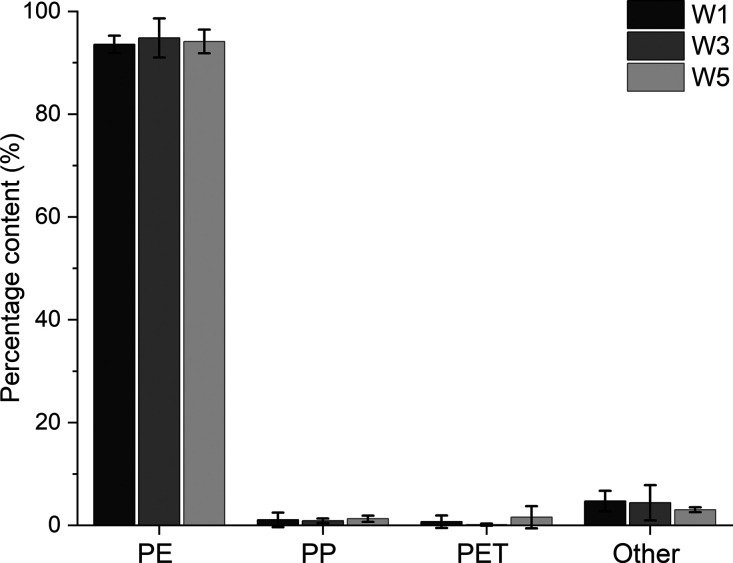
Weight proportions of different plastics in the PE-2D fractions sampled in three different weeks.

The percentages by weight of the different plastics found in the PE-2D fractions sampled in the three different weeks are shown in Figure 3. *W*_1_ and *W*_3_ are the averages of three successive days of sampling, while *W*_5_ is the average of 2 days. The bars show the standard deviations. The other polymers (indicated in Figure 3) included PS, PVC, and waste materials such as textile and paper.

Figure 3 shows that there was no significant variation in the PE content of the PE-2D fraction, either between different days in the week or between different weeks during the sampling period. The purity ratio based on all samples was 94 ± 2 wt % at a 95% confidence level. The contamination by PP in the PE fraction was about 1 wt %, as can be seen elsewhere.^24,25^

**Figure 4:**
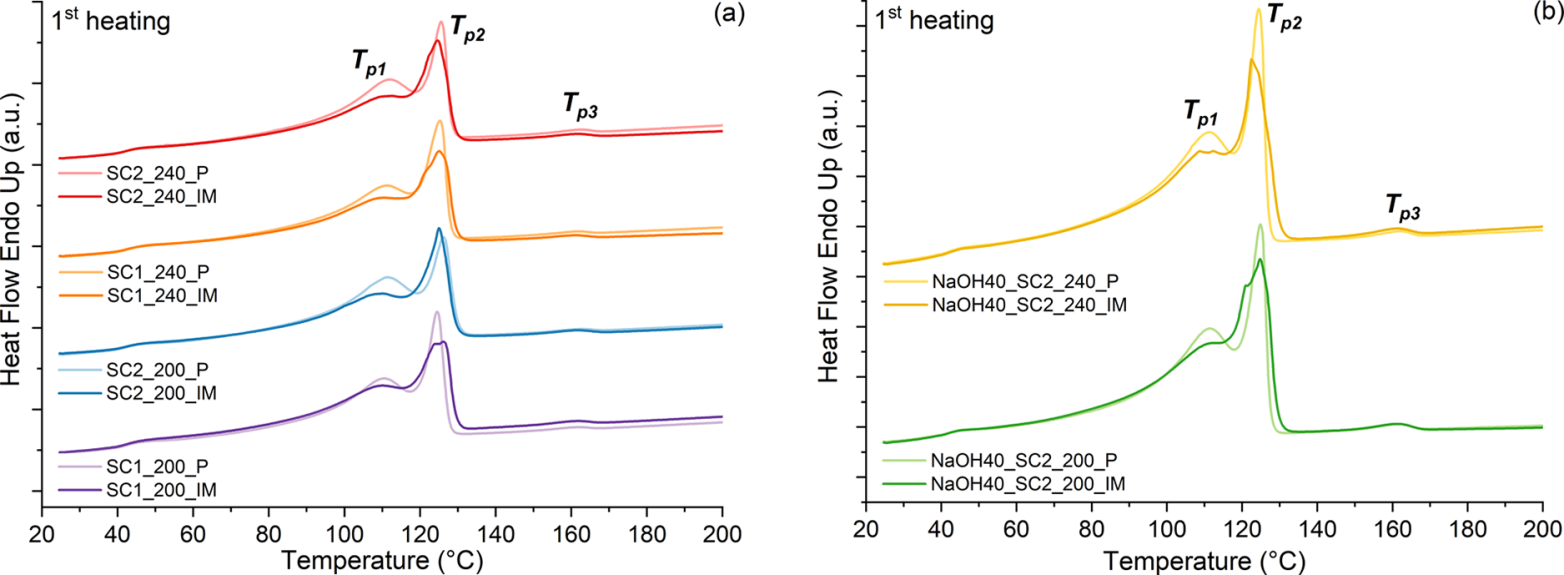
First heating curves of (a) unwashed samples and (b) washed samples. The light colors show the characterization of pellets (P) after compounding and dark colors the characterization of injection-molded samples (IM).

The first heating curves of the pellets (P) and of the injection-molded samples (IM) are presented in Figure 4.

In Figure 4, three melting peaks can be observed at 110, 125, and 161 °C, given in more detail in Table 2. The first melting peak (*T*_p1_) was related to the LDPE with a melting temperature of typically 105–118 °C,^26^ and the second (*T*_p2_) was related to the combination of different grades of PE, namely, MDPE, LLDPE, and HDPE, typically at 120–125, 126, and 126–135 °C, respectively.^26^ The third (*T*_p3_) much smaller peak was related to PP.^26^ The effects on the melting temperatures of screw configuration, compounding temperature, and washing were negligible. However, there was a slight increase in heat of fusion (Δ*H*) of the samples after washing, probably due to an increased crystallization as a result of a reduced content of impurities after washing.^27^

**Table 2:** Thermal Properties of the Samples^a^

	*T*_p1_ (°C)		*T*_p2_ (°C)		*T*_p3_ (°C)		Δ*H* (J/g)		*T*_ox_ (°C)		
sample	P	IM	P	IM	P	IM	P	IM	P	IM	ash content at 550 °C (%)
SC1_200	111	110	125	126	161	161	76	73	222	222	5.4
SC2_200	112	110	127	125	162	161	78	72	224	224	4.8
SC1_240	112	110	126	126	161	161	76	70	216	214	5.2
SC2_240	112	112	126	125	162	161	77	72	216	217	4.8
NaOH40_SC2_200	112	113	125	123	161	161	86	89	211	214	3.5
NaOH40_SC2_240	111	110	125	123	162	161	94	89	210	211	3.3

a P: pellets after compounding, IM: injection-molded samples.

Table 2 also shows the oxidation temperatures and ash contents. The oxidation temperature of both pellets and IM samples was slightly higher at a lower temperature of compounding, but the screw design had no effect. There was a slight decrease after washing, but all the samples had a *T*_ox_ of at least 210 °C, implying that active stabilizers remained in the samples, since the *T*_ox_ for the unstabilized virgin PE has typically been reported as being 180 ± 5 °C.^28^ These minor decreases in *T*_ox_ values indicate a degradation by increased compounding temperature and by washing but do not provide any information about the degradation mechanism itself.

An ash content in the unwashed material of about 5% was expected, and the simple washing reduced the ash content to about 3%, also as expected.^5^

The weight-average molecular mass (*M*_w_) of the pellets made of unwashed material (sample SC2_240) was 115 500 g/mol with a PDI of 4.5, and washing resulted in a slight decrease. The NaOH40_SC2_240 sample had a *M*_w_ of 114 500 g/mol with a PDI of 4.6. This decrease in *M*_w_ after washing corresponded to the slight reduction in *T*_ox_. The recycled samples, as shown in DSC thermograms, contain a mixture of different grades of PE and traces of other polymers, mainly PP. It is known that two mechanisms of degradation, chain scission and chain branching or crosslinking, occur simultaneously, the former being dominating for HDPE, LLDPE, and PP, while the latter for LDPE; thus, the heterogeneity of the samples restricts to state which mechanism prevails here since the differences are quite small.^29−33^ Moreover, there might be an additional influence on degradation by the NaOH washing considering the resulted alkali residues as reported in another study.^23^

The materials compounded at 200 °C regardless of screw design had MFR values of 0.7 g/10 min while those compounded at 240 °C had 0.8 g/10 min. Similarly, after washing, the MFR values were 0.7 and 0.6 g/10 min for the samples compounded at 200 and 240 °C, respectively. Compounding at a higher temperature resulted consistently in a slightly lower viscosity for the unwashed material than for the washed material, but after washing, there was a slight increase in viscosity for the sample compounded with SC2 at 240 °C. The MFR values all lie in the range typical for a blown film, pipe extrusion, extrusion blow molding, or toward the lower end of the values for injection-molded virgin materials, indicating that packing films, shrink films, flexible bottles, and monofilaments could probably be made of recycled materials.^34^

**Figure 5:**
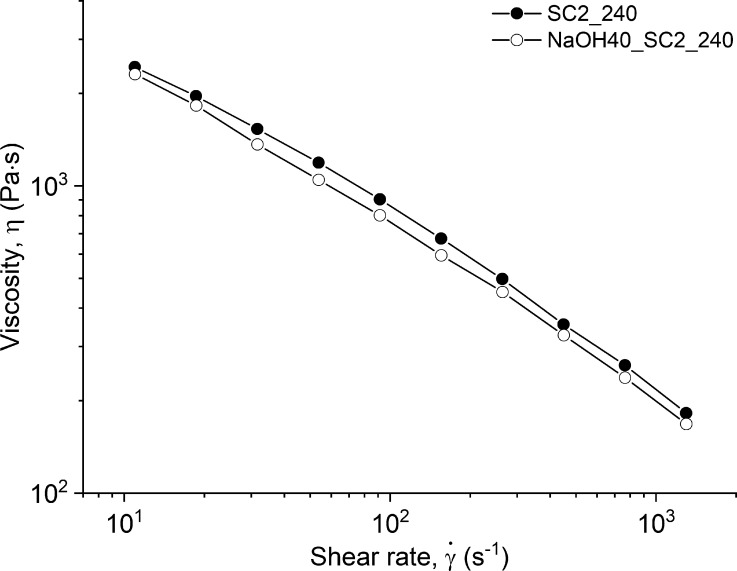
Viscosity as a function of shear rate for the unwashed and washed pellets.

The flow curves of the samples compounded using SC2 at 240 °C, before and after washing, are presented in Figure 5.

The curve of viscosity versus shear rate shows a minor decrease in viscosity after washing. This decrease is not shown in MFR, possibly due to MFR measurement being less well defined in terms of viscosity and shear rate. As it is reported in the literature, chain branching, due to degradation, causes the viscosity to increase, *M*_w_ to decrease, and PDI to increase, whereas chain scission leads to a lower viscosity and decreased *M*_w_; thus, the complexity of the studied material prohibits to define the dominating mechanism, and the disagreement between two characterizations remains unclear.^35−39^ As suggested by Santana and Gondim,^23^ this may be an effect of washing parameters such as washing agent and temperature as well as drying conditions on the degradation of PE. The small reduction in *M*_w_ after washing may explain the slight decrease in viscosity shown in Figure 5.

**Figure 6:**
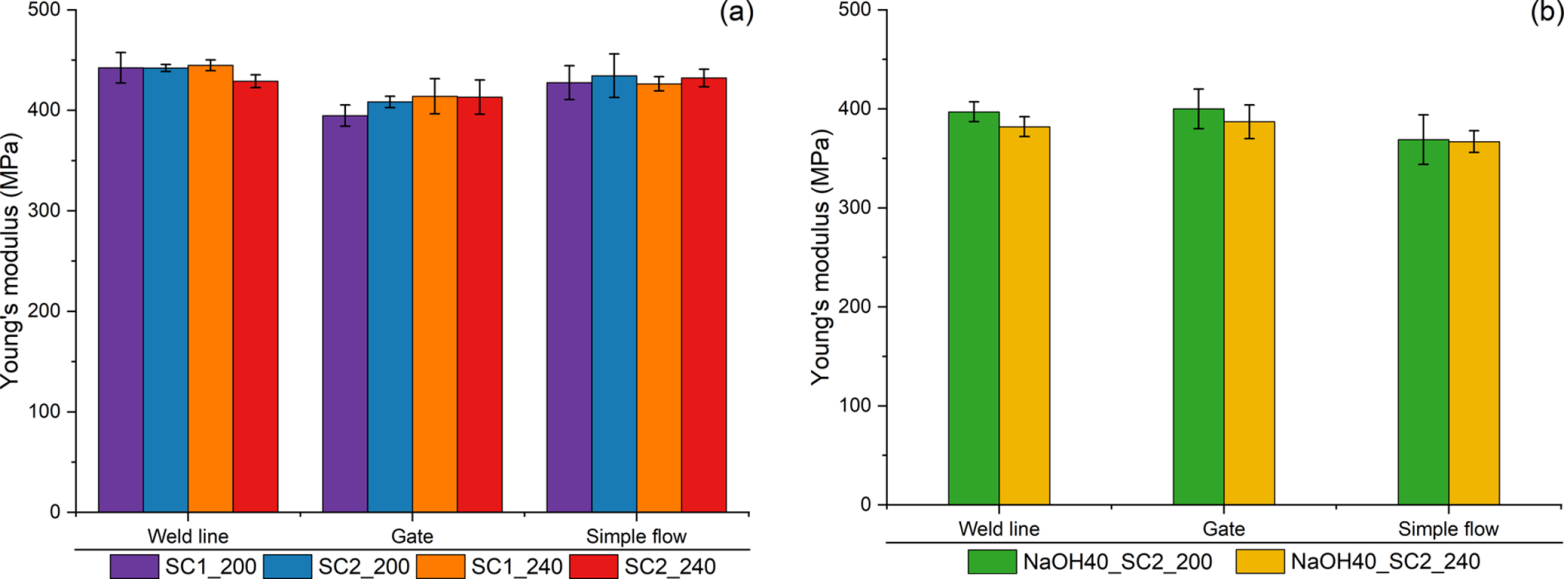
Young’s modulus of the samples at different regions in the IM frame: (a) unwashed and (b) washed.

Figure 6 shows the Young’s modulus values for different screw designs (SC1 and SC2) and different compounding temperatures (200 and 240 °C) for the three positions in the injection-molded samples.

The average values of the modulus for the unwashed samples, Figure 6a, varied between 429–445, 395–415, and 426–434 MPa for the weld line, gate, and simple flow regions, respectively. Neither the parameters during compounding nor the structure of the molded sample had a major influence on the tensile Young’s modulus. The effect of washing was small. Figure 6b shows that the average Young’s modulus varied between 367 and 400 MPa, considering all the test sections. The results were higher than the average value for recycled PE film (rPE_film_) grades reported by Demets et al.^34^ and may be sufficiently high for applications such as injection-molded, blow-molded products.

**Figure 7:**
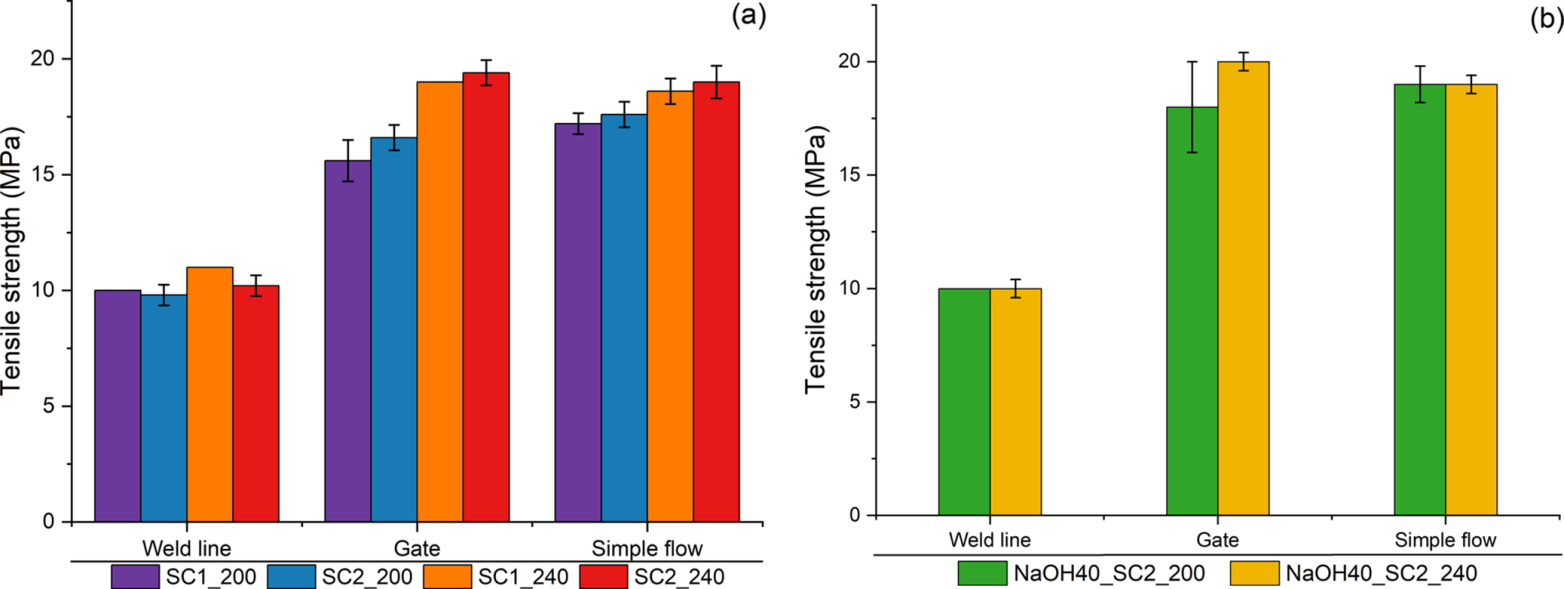
Tensile strength of samples taken from different regions of the IM frame: (a) unwashed and (b) washed.

Figure 7 shows the tensile strength of molded samples made with different screw designs (SC1 and SC2) of different compounding temperatures (200 and 240 °C), taken from the different regions of the injection-molded samples.

As expected, the bars with the weld line had the lowest tensile strength of ca. 10 MPa for all samples. In the other two regions, that is, the gate and the simple flow samples, the strength was 15–20 MPa for the unwashed and ca. 20 MPa for the washed material with a slight increase with compounding at the higher temperature (240) and the higher shear screw configuration (SC2), possibly due to melting of polymer contaminants which otherwise might act as stress concentrators.^40^ The strength at the weld lines was unexpectedly high, close to the reported value (13 MPa) for virgin PE-LLD, in all molded samples,^41^ but it should be kept in mind that the samples used in these papers had different dimensions. The strength measured in the gate and in the simple flow regions had values similar to the reported average value of rPE_film_ grade.^34^

**Figure 8:**
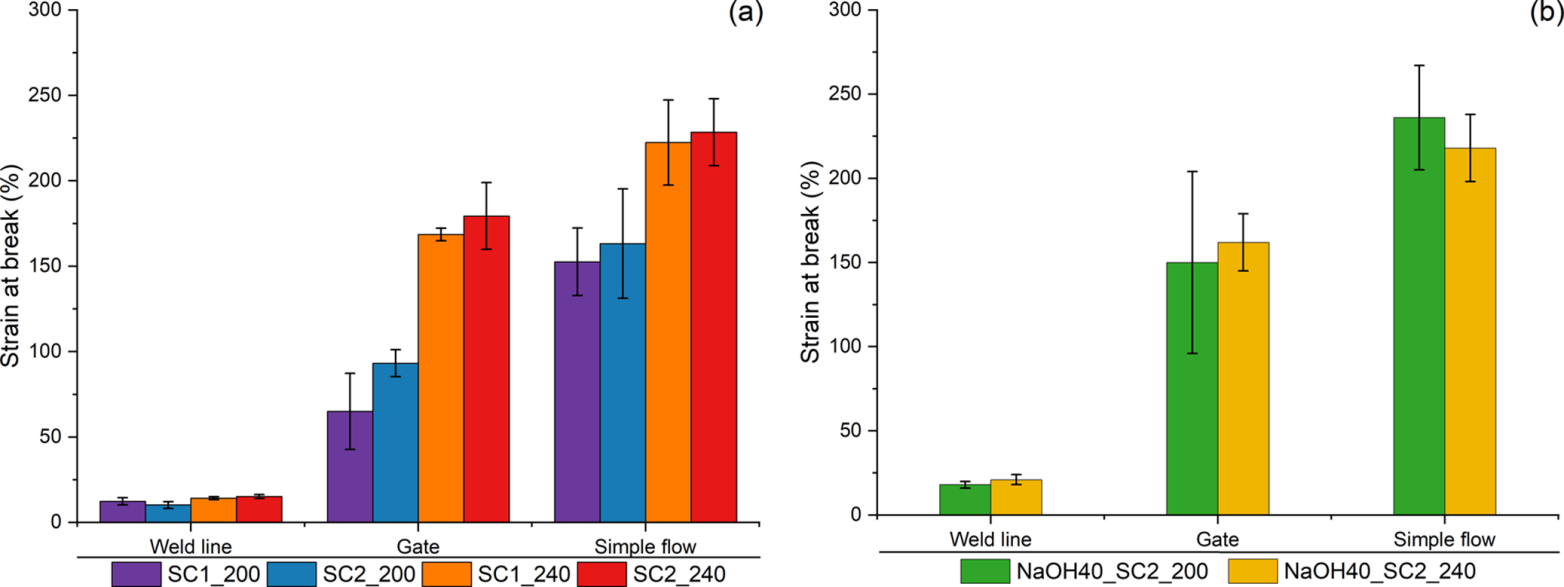
Strain at break of samples taken from different regions of the IM frame: (a) unwashed and (b) washed.

Figure 8 shows the strain at break of molded samples. The weld line regions showed the lowest strain at break for both unwashed and washed samples in the range of 10–15 and 15–20%, respectively. The simple flow region had higher strains at break (150–230% for unwashed and 220–240% for washed) than the gate regions (65–180% for unwashed and 150–160% for washed), and in both the gate and simple flow regions, the unwashed samples have higher strain values with increasing processing temperature and higher mixing (SC2). The influence of temperature at compounding seems to be more important than the screw design, and in the case of lower temperature, there is a risk of more unmolten plastics that could act as a stress concentrator leading to an earlier break.^40^ The opposite effect was observed with increasing compounding temperature for the washed samples especially in the simple flow region. With washing due to the applied float–sink separation, the heavier polymer fractions were mostly eliminated, which decreased the chance of having unmolten plastics acting as a stress concentrator especially with lower compounding temperature. Additionally, temperature-induced degradation might prevail more with higher compounding temperature considering the cleaner material.^42^ When unwashed samples were compared with the corresponding washed samples, there is an increase in elongation at break after washing with lower compounding temperature whereas a slight decrease with higher compounding temperature, considering both the gate and simple flow regions. Overall, however, washing led to a slight increase in strength and in strain at break probably due to less contamination and other molecular changes.^5,7,33^ The strain at break values, with the exception of those in the weld line regions, were close to the average value reported for rPE_film_ but lower than the values reported for virgin grades of PE for applications in films or flexible IM products.^34^ Since simple washing increased the strain at break, it may be interesting to explore washing parameters further.

**Figure 9:**
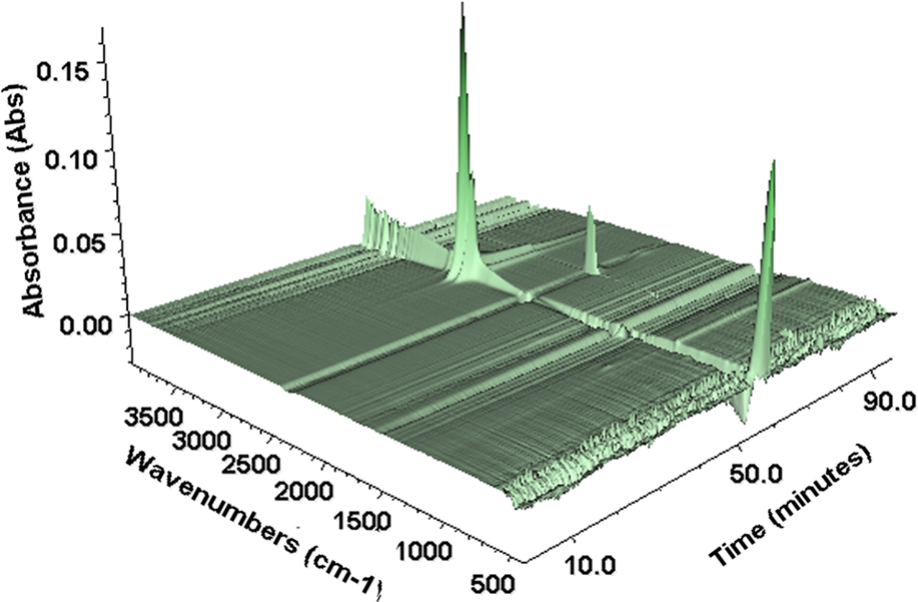
3D simulations of the time-resolved IR spectra at TGA/FTIR characterization of the unwashed material compounded with SC1 at 200 °C (switch from N_2_ to air at 72.5 min).

**Figure 10:**
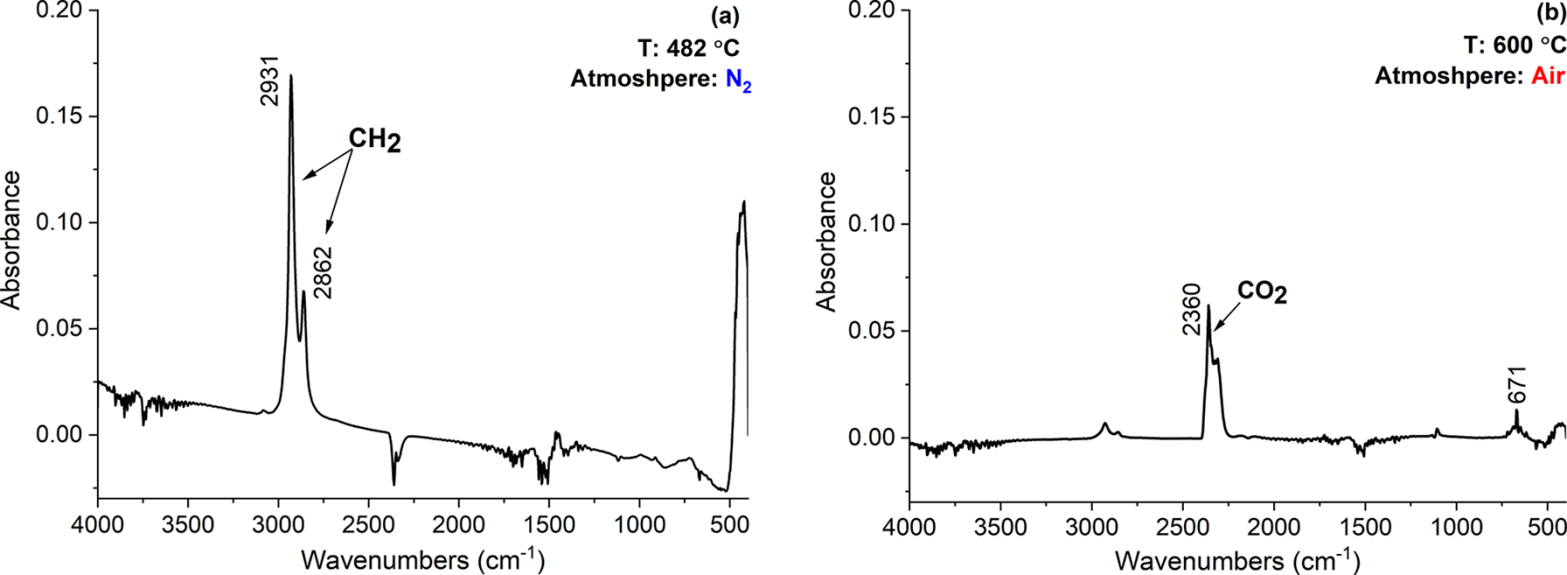
Individual FTIR spectra chosen from 3D simulation showing the maximum absorption peaks located at a certain time/temperature of the analysis in (a) N_2_ at 56 min and (b) air after 73 min.

For pellets produced of the unwashed materials compounded with SC1 at 200 °C, the gases evolved during heating were analyzed by FTIR spectroscopy, as shown in Figures 9 and 10.

Figure 10a presents the IR spectrum at 56 min/482 °C, when the absorption peaks of CH_2_ groups at 2931 and 2862 cm^–1^ related to the PE reached their maximum intensities.^43^ Switching the gas to air immediately led to a CO_2_ peak at 2360 cm^–1^,^44^ at 73 min/600 °C, as shown in Figure 10b. The main degradation product in the air was CO_2_, which is usual, and mononuclear aromatic hydrocarbons (C–H bending) were also evolved at 671 cm^–1^ with time. The OMNIC spectra results showed that the spectrum (Figure 10a) indicated traces of amines or alkanes which are common volatile organic compounds in plastics, often causing the odor found in the post-consumer recycled material.^45^
